# Changes in Neuronal Excitability by Activated Microglia: Differential Na^+^ Current Upregulation in Pyramid-Shaped and Bipolar Neurons by TNF-α and IL-18

**DOI:** 10.3389/fneur.2016.00044

**Published:** 2016-03-30

**Authors:** Lars Klapal, Birte A. Igelhorst, Irmgard D. Dietzel-Meyer

**Affiliations:** ^1^Department of Biochemistry II, Ruhr-University Bochum, Bochum, Germany

**Keywords:** voltage-activated sodium currents, microglia, hippocampal neurons, interleukin-18, tumor necrosis factor-α, lipopolysaccharide, transforming growth factor-β, glial cells

## Abstract

Microglia are activated during pathological events in the brain and are capable of releasing various types of inflammatory cytokines. Here, we demonstrate that the addition of 5% microglia activated by 1 μg/ml lipopolysaccharides (LPS) to hippocampal cultures upregulates Na^+^ current densities (I_NavD_) of bipolar as well as pyramid-shaped neurons, thereby increasing their excitability. Deactivation of microglia by the addition of 10 ng/ml transforming growth factor-β (TGF-β) decreases I_NavD_ below control levels suggesting that the residual activated microglial cells influence neuronal excitability in control cultures. Preincubation of hippocampal cultures with 10 ng/ml tumor necrosis factor-α (TNF-α), a major cytokine released by activated microglia, upregulated I_NavD_ significantly by ~30% in bipolar cells, whereas in pyramid-shaped cells, the upregulation only reached an increase of ~14%. Incubation of the cultures with antibodies against either TNF-receptor 1 or 2 blocked the upregulation of I_NavD_ in bipolar cells, whereas in pyramid-shaped cells, increases in I_NavD_ were exclusively blocked by antibodies against TNF-receptor 2, suggesting that both cell types respond differently to TNF-α exposure. Since additional cytokines, such as interleukin-18 (IL-18), are released from activated microglia, we tested potential effects of IL-18 on I_NavD_ in both cell types. Exposure to 5–10 ng/ml IL-18 for 4 days increased I_NavD_ in both pyramid-shaped as well as bipolar neurons, albeit the dose–response curves were shifted to lower concentrations in bipolar cells. Our results suggest that by secretion of cytokines, microglial cells upregulate Na^+^ current densities in bipolar and pyramid-shaped neurons to some extent differentially. Depending on the exact cytokine composition and concentration released, this could change the balance between the activity of inhibitory bipolar and excitatory pyramid-shaped cells. Since bipolar cells show a larger upregulation of I_NavD_ in response to TNF-α as well as respond to smaller concentrations of IL-18, our results offer an explanation for the finding, that in certain conditions of brain inflammations periods of dizziness are followed by epileptic seizures.

## Introduction

Glial cells influence neuronal functions in various ways. Apart from preventing mutual excitation of adjacent neurons by K^+^ buffering and glutamate uptake (1), they can also modulate neuronal excitability. For instance, thyroid hormone triiodo-l-thyronine (T3) upregulates Na^+^ currents and thereby increases excitability of postnatal rat hippocampal and cortical neurons (2, 3) via secretion of proteins [most prominently basic fibroblast growth factor (FGF-2)] from satellite cells (4).

Microglia are the resident macrophage-like population in the central nervous system (CNS) (5–7). They are continually active in their resting state, palpating and surveying their microenvironment with extremely motile processes (8). Pathological events lead to a rapid change of the microglial morphology (9) and the expression of cell surface proteins (10) such as antigens (11). A well-established method of activating microglia in culture is their stimulation via lipopolysaccharides (LPS) (12), the major surface membrane components of Gram-negative bacteria. The LPS stimulus on microglia is mediated by Toll-like receptors 1–9 (TLR), especially TLR2 (13). TLRs are a major family of pattern recognition receptors that mediate innate immunity but also link with the adaptive immune response, therefore providing a mechanism by which microglia are able to sense both pathogen- and host-derived ligands within the CNS (14, 15). In this activated state, microglia secrete a variety of cytokines (16), including proinflammatory factors like the IL-1/IL-18 family members and TNF-α.

Some growth factors and cytokines do not only regulate cell survival, differentiation, and proliferation but are also able to modulate the expression of ion channels. Long-term effects on the expression of Na^+^ channels by nerve growth factor (NGF) have already been shown in DRG neurons (17, 18) and nociceptive primary afferent neurons (19). Likewise, FGF-2 upregulates Na^+^ current density in PC12 cells (20) as well as hippocampal neurons (4). Recently, TNF-α has been shown to contribute to the upregulation of Nav1.3, Nav1.8 (21), and Nav1.7 (22) in dorsal root ganglion (DRG) neurons (21) and to enhance Na^+^ currents in injured DRG neurons (18). Since the voltage-gated sodium channels are essential for the generation and conduction of electrical impulses in excitable cells, an upregulation of the Na^+^ current density (I_NavD_) especially of Nav1.7 and Nav1.8 has been suggested to be involved in the enhancement of excitability in inflammation in the peripheral nervous system (23–27) leading to hyperalgesia, inflammatory, and neuropathic pain.

In the CNS, inflammations are accompanied by conditions ranging from dizziness to epileptic seizures (28). In infectious diseases, transient epileptic episodes have been observed (29, 30), which have been suggested to be caused by excessive cytokine release from activated microglia (31, 32). Epileptiform neuronal excitability can be induced by changes in the intrinsic neuronal excitability governed by the membrane density of voltage-activated sodium channels (Nav1.1–Nav1.3 and Nav1.6) leading to a stronger increase of excitation compared with inhibition in the neuronal network. We have recently obtained the first evidence that in hippocampal cultures, the addition of 5% microglia stimulated by 1 μg/ml LPS as well as an incubation of the cultures with 100 ng/ml TNF-α leads to an upregulation of I_NavD_ (33). As in the conditions of neuroinflammation in the CNS periods of dizziness may be followed by epileptic events (34) and TNF-α can suppress cortical excitability (35), it is intriguing to approach the question of whether activated microglia could regulate I_NavD_ differentially in pyramid-shaped (excitatory) and bipolar (inhibitory) neurons. The main aim of this study was to investigate whether both types of neurons regulate I_NavD_ differently in the presence of activated microglia, whether different TNF-α receptor subtypes are involved in the response of bipolar and pyramid-shaped cells, and whether additional cytokines, which are released from microglia, such as interleukin-18 (36), could participate in the regulation of I_NavD_ in both cell types. In order to obtain a first evidence whether already the presence of residual microglia in control cultures influences I_NavD_, we additionally investigated whether an inhibition of microglia by transforming growth factor β (TGF-β) (37) influences I_NavD_.

## Materials and Methods

### Cell Cultures

Experiments were performed on cell cultures obtained from hippocampi dissected from brains of 2–4 days postnatal Wistar–Hannover rats. The hippocampi were collected in ice-cold modified phosphate buffered saline (MPBS) [0.89 mM KH_2_PO_4_, 2.7 mM KCl, 5 mM Na_2_HPO_4_, 137 mM NaCl, 10 mM glucose, 10 mM HEPES, 1 mM pyruvate, 1 mM glutamine, 1 mg/ml bovine serum albumin (BSA), 25 U/ml penicillin, and 25 μg/ml streptomycin (P/S from PAA, Germany)]. After addition of 10 μg/ml desoxyribonuclease I and 5 μl/ml of a 2.5% trypsin solution and gentle dissociation, the tissue was incubated under agitation for 7 min at 37°C. The digestion was stopped with 50 μl/ml fetal calf serum (FCS, Invitrogen, Karlsruhe, Germany) and afterward triturated 15 times with a 1-ml Eppendorf pipette tip. The dissociated cells were collected in a 10-ml tube and centrifuged at 1,000 rpm at 4°C for 10 min. The pellet was resuspended in RPMI (Invitrogen, Karlsruhe, Germany), supplemented with 10% FCS, P/S, and glutamine. The cells were preplated at 37°C and 5% CO_2_ in a humidified atmosphere for 1 h in a B 5060 incubator (Heraeus, Hanau, Germany). After preplating, the neuron-enriched supernatant was collected and centrifuged at 1,000 rpm at room temperature for 10 min. The pellet was resuspended in supplemented RPMI medium. The 3.5-cm plastic petri dishes were coated with poly-d-lysine (5 μg/ml in sterile water, for 1 h) and 300,000 cells of the suspension were transferred into 1-cm diameter glass rings in the center of the dishes. In some experiments, ~15,000 microglia were added directly to the cells in the central glass ring. To expose neurons to microglia-secreted factors in the absence of direct membrane contact 60,000 microglia were transferred into cell culture inserts (DIM 23/34, 0.4 MY) (Fisher scientific, Schwerte, Germany). Since the inserts had a diameter of 2.4 cm (four times the surface of the glass rings) four times as many microglia were seeded to achieve a comparable density of microglia directly above the neurons.

After culturing for 1 day, the medium was exchanged with Neurobasal medium (NB) supplemented with B18 prepared according to the composition described in Ref. (38) without the thyroid hormone triiodo-l-thyronine (T3) (to minimize potential influences of astroglia-secreted factors on Na^+^ current densities) and treated for 24 h with 4 μM Cytosin-β-d-Arabinofuranoside (AraC) to prevent further proliferation of glial cells and subsequent overgrowth of the cultures. After the third day in culture, the NB-medium was renewed and supplemented with the factors to be investigated, 10 ng/ml TGF-β (Sigma Aldrich, Munich, Germany), 10–100 ng/ml TNF-α (Peprotech, Hamburg, Germany), 1 μg/ml LPS, 5, 8 or 10 ng/ml IL-18 (Invivogen, CA, USA), and/or 10 μg/ml anti-TNFR 1/2 (R&D-Systems, MN, USA) (as indicated in the figures).

### Isolation of Microglia

Microglial cells were obtained from whole brain mixed glial cell cultures by a shaking procedure following the protocol published by McCarthy et al. (39) and modified as described by Kleinsimlinghaus et al. (40). Cortices were dissected from 0 to 3 days postnatal Wistar–Hannover rats and dissociated by passing through 125- and 36-nm nylon meshes. The cells were centrifuged at room temperature at 900 rpm for 10 min and resuspended in 5 ml glial mixed medium (GMM) composed of DMEM:Ham’s F12 (1:1) supplemented with 10% FCS, P/S, and glutamine. The cells harvested from 1.5 brains were transferred to uncoated T-75 flasks (Sarstedt, Nümbrecht, Germany) and precultured for 10–12 days in GMM at 37°C, 5% CO_2_, and a humidified atmosphere in an incubator (B 5060, Heraues, Hanau, Germany) with medium changes every 3–4 days.

Following the preculture period, microglia were isolated from the underlying layers of stronger adhering astrocytes and oligodendrocyte precursor cells by shaking the flasks for 3 h on an orbital shaker (ES-W, Kühner AG, Birsfelden, Switzerland) in the incubator. The supernatant containing more than 90% microglia was centrifuged for 5 min at 1,000 rpm at room temperature and added to the neuron-enriched cultures.

### Patch-Clamp Recordings

Na^+^ currents were quantified using whole cell patch clamp recordings. All measurements were performed at room temperature using a Patch-Clamp L/M-EPC7 amplifier (List Medical, Darmstadt, Germany). The maximal measurement period for one cell culture dish was limited to 60 min since the number of successful recordings with small leakage currents decreased due to deteriorating cells. Signal filtering was performed using the EPC7 10-kHz lowpass filter and data obtained were digitized using PClamp 10 software (Molecular Devices, Sunnyvale, CA, USA) at a sampling rate of 20 kHz. Data were digitized with a Digidata 1440A board (Molecular Devices) and stored on a personal computer. Patch pipettes were fabricated from borosilicate glass capillaries (GB-150TF-8P, Science Products, Hofheim, Germany) using a PP-830 puller (Narishige Europe, London, UK) and had resistances of 6–10 MΩ. The pipette solution contained in mM: CaCl_2_ 0.1, EGTA 1.1, MgCl_2_ 5, NaCl 5, HEPES 10, and CsF 100; The bath solution contained in mM: CdCl_2_ 0.5, CaCl_2_ 1, MgCl_2_ 1, 4-Aminopyridine 4, glucose 10, HEPES 10, TEA-Cl 10, and NaCl 100. The osmolarity of bath and pipette solution was adjusted to the osmolarity of the NB medium (40).

Na^+^ currents were recorded using a series of step depolarizations, starting from a holding potential of −77 mV (after a liquid junction potential correction of −7 mV) in increments of 5 mV. Maximal peak Na^+^ currents were determined as peak currents at a test potential of −12 mV, which corresponds to the maximum of the current–voltage relationship.

Voltage-dependent inactivation of the Na^+^ currents was determined by applying a series of prepulse steps of 200 ms duration in 5 mV increments starting at an initial hyperpolarization to −82 mV, which were followed by a depolarization step to a test potential of −17 mV to evoke maximal Na^+^ currents. Peak Na^+^ currents versus prepulse potential were fitted to a modified Boltzmann equation [*I*/*I*_0_ = 1/(1 + exp([*V*_m_ − *V*_In1/2_]/S)) *V*_In1/2_ = prepulse potential at which half of the channels are inactivated; *I*_0_ = current elicited from the most negative prepulse potential; *S* = slope factor].

Leakage and capacitive artifacts were subtracted using a P/4 protocol. By normalizing the peak Na^+^ current to the cell capacitance (calculated from the integral of the charging curve), Na^+^ current densities were calculated. Cells were only included in the statistical evaluation if their leak currents did not surpass 100 pA; their series resistances did not surpass 20 MΩ and Na^+^ currents displayed an activation voltage range of at least 20 mV in the *I*/*V* curve to minimize errors of poor membrane voltage control. A liquid junction potential of −7 mV with respect to the bath solution was corrected manually. For statistical comparison, analysis of variance (ANOVA) followed by Tuckey’s *Post hoc* test was used (OriginPro 9.0G, Origin Lab Corporation, Northampton, MA, USA), as it represents a conservative approach when sample sizes are not equal, and we expect homoscedasticity, normal distribution, and independence of our data.

## Results

### Regulation of Na^+^ Current Density by Microglial Activation and Deactivation

**Figure 1:**
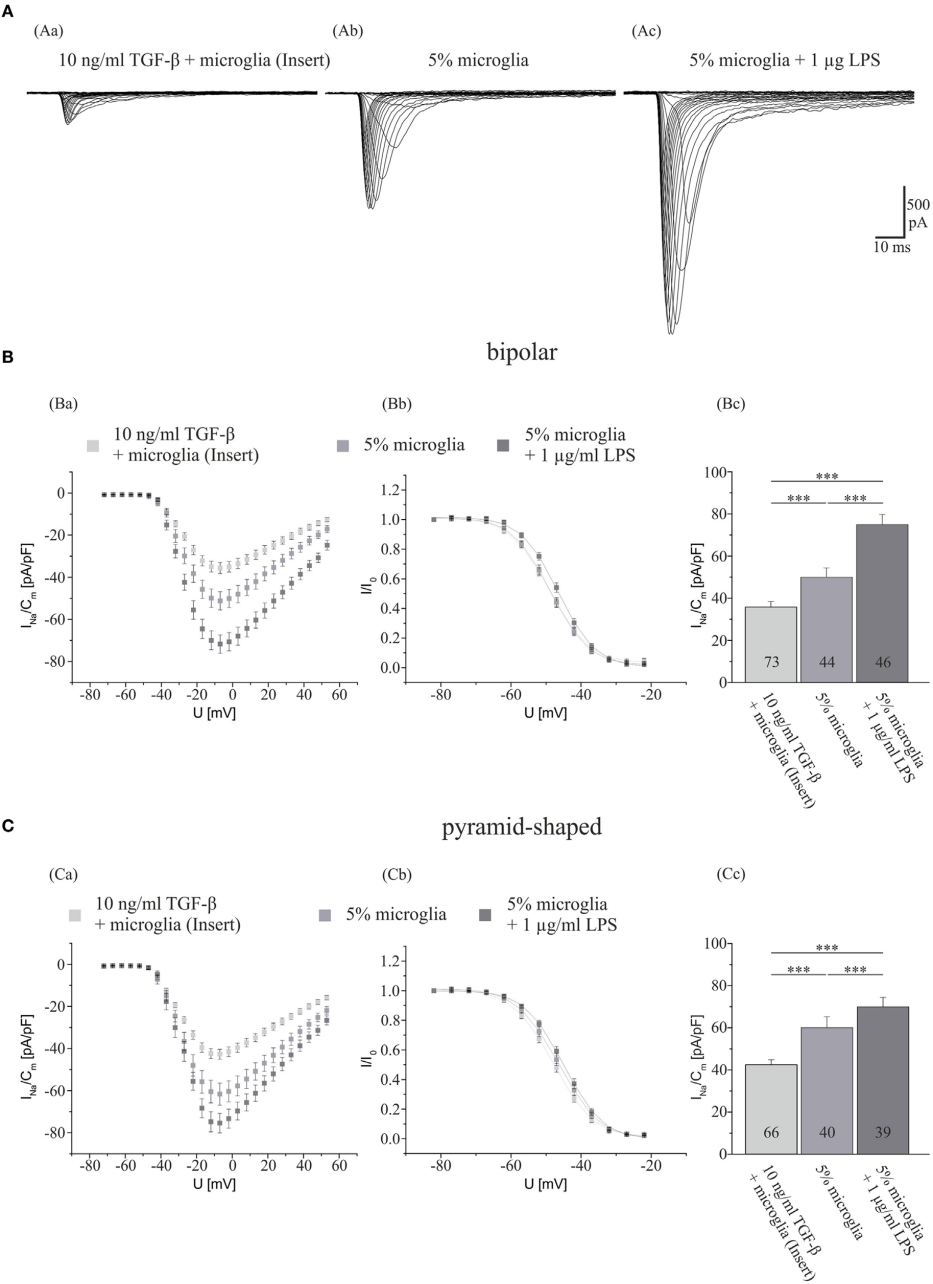
**Influences of LPS exposure for 4 days and TGF-β exposure for 7 days in the presence of microglia on Na^+^ current density of cultured hippocampal neurons**. **(A)** Series of original Na^+^ current recordings from bipolar neurons elicited by step depolarizations in 5 mV increments starting from a holding potential of −77 mV after the different treatments. Analysis of the whole data set for **(B)** bipolar and **(C)** pyramid-shaped cells. (Ba,Ca) Average current–voltage relationships for Na^+^ currents normalized to capacitance recorded from neurons cultured in the presence of microglia and either 4 days of exposure to 1 μg LPS or 7 days of exposure to TGF-β. (Bb,Cb) Influence of TGF-β and LPS on steady-state inactivation of Na^+^ currents. Solid lines represent fits to the modified Boltzman equation as detailed in Section “Materials and Methods.” (Bc,Cc) Peak Na^+^ current densities of neurons cultured in the presence of microglia and either 4 days of exposure to 1 μg/ml LPS or 7 days of exposure to TGF-β determined at test potentials of −12 mV starting from holding potentials of −77 mV. All recordings performed at day 7 in culture. Numbers in bar charts indicate numbers of cells recorded from. Database for every column derived from 7 to 10 different preparations. Error bars represent means ± SE, *p < 0.05, **p < 0.01, and ***p < 0.005.

We first investigated, whether a cocultivation of hippocampal neurons with a surplus of either activated or deactivated microglia for 7 days influences their Na^+^ current density. To this aim, control Na^+^ currents were measured in hippocampal cultures obtained from 2- to 4-day-old postnatal rats cocultivated with ~15,000 (5%) microglia. Currents recorded from control neurons after 7 days in culture were compared with those recorded in sister cultures in which microglia had been activated by addition of 1 μg/ml LPS after the first 3 days in culture. In a third series of dishes, the influence of microglia on neurons was abolished as follows: to suppress direct membrane interactions between microglia and neurons, instead of adding microglial cells directly, the same density of microglia was separated from the neurons by seeding into inserts. To inactivate protein secretion from microglia 10 ng/ml TGF-β was added in every medium exchange throughout the whole culture period of 7 days. As shown in Figure 1, activation of microglia via LPS treatment resulted in a significant increase in Na^+^ current density in both bipolar and pyramid-shaped cells increasing I_NavD_ in bipolar cells from 49.9 ± 4.4 pA/pF (mean ± SE) to 69.9 ± 4.5 pA/pF in LPS-stimulated cultures (*p* < 0.005). Likewise, the Na^+^ current density increased from 60.2 ± 5.2 to 74.8 ± 4.8 pA/pF in pyramid-shaped neurons (*p* < 0.005). The suppression of microglial activation by 10 ng/ml TGF-β led to a significant decrease in Na^+^ current density to 34.9 ± 2.5 pA/pF (bipolar, *p* < 0.005) and 42.4 ± 2.4 pA/pF (pyramid-shaped, *p* < 0.005). This suggests that the degree of microglia activation in the vicinity of neurons considerably influences neuronal Na^+^ current density. Additionally, the Na^+^ current densities of bipolar and pyramid-shaped neurons were significantly different in cultures incubated with 10 ng/ml TGF-β (*p* < 0.01). As shown in Figures 1Bb,Cb, no changes in voltage dependence of activation and inactivation were observed.

### Effects of Neuroactive Factors Derived from Activated Microglia

#### Effect of TNF-α on the Na^+^ Current Density

**Figure 2:**
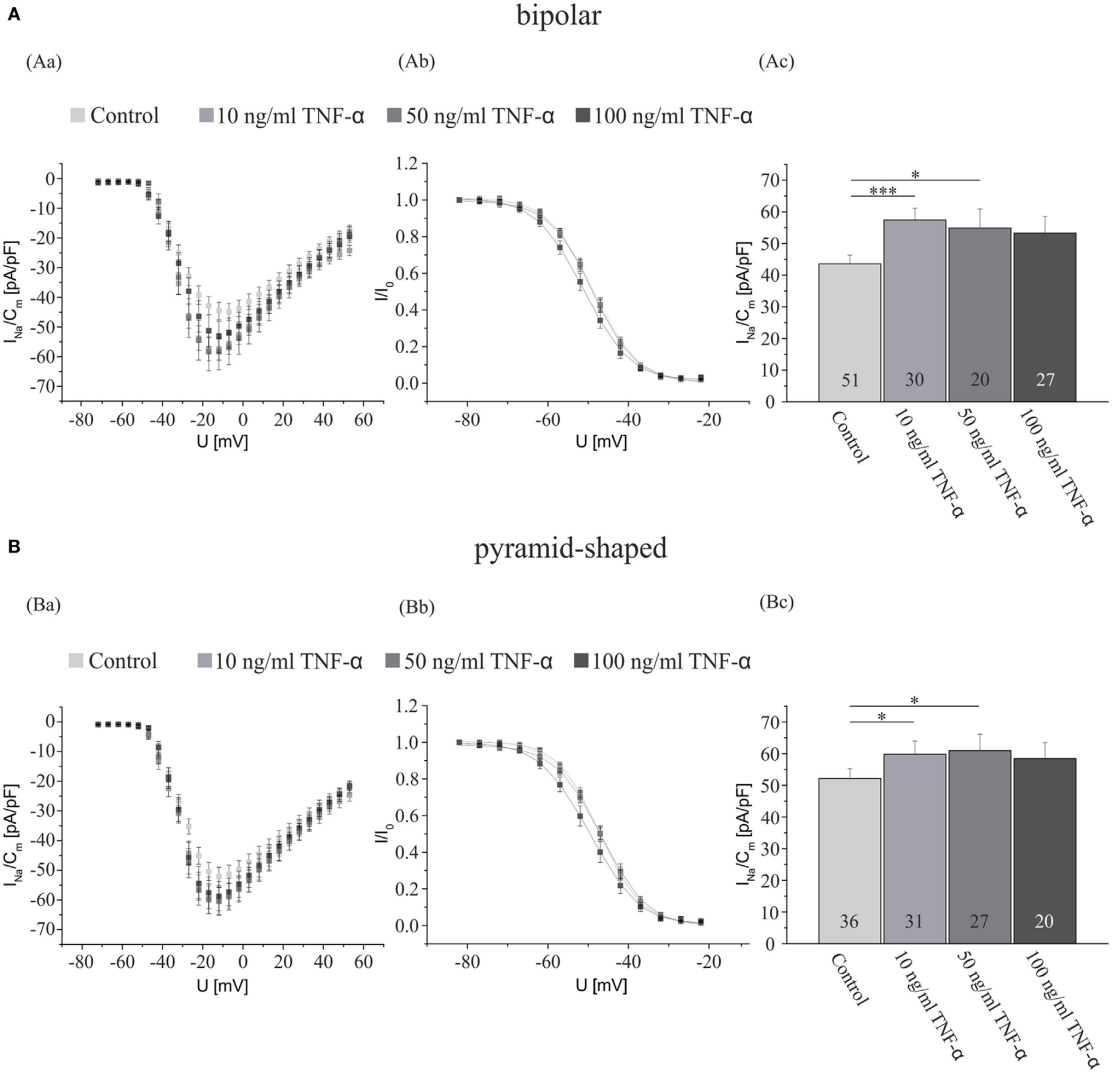
**TNF-α exposure for 4 days increases the Na^+^ current density of cultured hippocampal neurons**. Analysis of the whole data set for **(A)** bipolar and **(B)** pyramid-shaped cells. (Aa,Ba) Average current–voltage relationships for Na^+^ currents normalized to capacitance recorded from neurons cultured in the presence or absence of 10, 50, or 100 ng/ml TNF-α for 4 days. (Ab,Bb) Influence of a preincubation with TNF-α on steady-state inactivation of Na^+^ currents. Solid lines represent fits to the modified Boltzman equation as detailed in Section “Materials and Methods.” (Ac,Bc) Peak Na^+^ current densities of neurons cultured in the presence or absence of 10, 50, or 100 ng/ml TNF-α for 4 days determined at test potentials of −12 mV starting from holding potentials of −77 mV. Note that maximal Na^+^ current densities are already measured at a concentration of 10 ng/ml of TNF-α, suggesting that the receptors are already saturated at this dosage. All recordings performed at day 7 in culture after incubations with test substances for 4 days following a preculture period of 3 days. Numbers in bar charts indicate numbers of cells recorded from. Database for every column derived from 4 to 10 different preparations. Error bars represent means ± SE, *p < 0.05, **p < 0.01, and ***p < 0.005.

After showing that activated microglia exert a considerable effect on the Na^+^ current density of hippocampal neurons, we now focused on identifying responsible neuroactive factors released from activated microglia. Since TNF-α regulates the Na^+^ current density in central (33) and peripheral DRG neurons (18, 21), we first recorded a dose–response curve of TNF-α effects on hippocampal neurons (Figure 2). Hippocampal neuron-enriched cultures obtained from 2- to 4-day-old postnatal rats were preincubated for 3 days and exposed to NB medium supplemented with or without (control) either 10, 50, or 100 ng/ml TNF-α. As the records in Figure 1 show for TGF-β treated cultures, in control cultures containing no surplus of microglial cells, current densities in bipolar neurons were significantly smaller than in pyramid-shaped neurons (43.6 ± 2.7 pA/pF in bipolar cells versus 52.2 ± 3.1 pA/pF in pyramidal cells, *p* < 0.05). A preincubation with all tested concentrations of TNF-α induced an increase in Na^+^ current density. A preincubation with 10 ng/ml significantly increased I_NavD_ in both neuron types, while 50 ng/ml led to a significant increase only in bipolar neurons. We observed a maximal increase in bipolar neurons by ~30% (from 43.6 ± 2.7 pA/pF, *n* = 51) and in pyramid-shaped neurons by ~15% (from 52.2 ± 3.1 pA/pF, *n* = 36). This suggests that the effect is already maximal at a concentration of 10 ng/ml.

#### Role of TNF-Receptor 1 and 2 in the Na^+^ Current Upregulation

**Figure 3:**
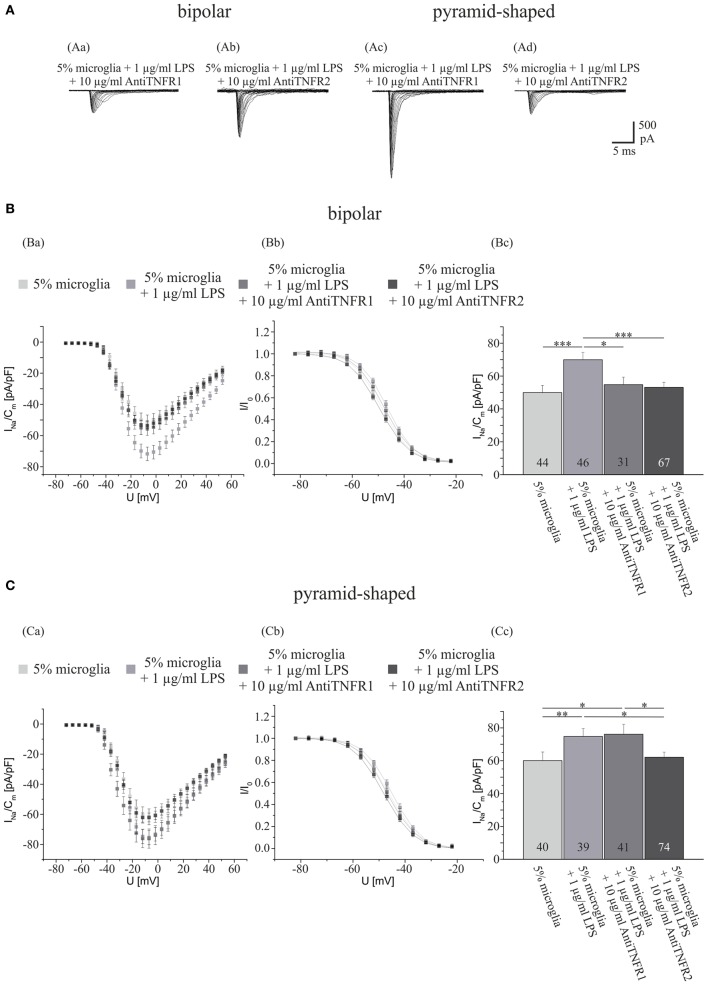
**Effects of preincubations with antibodies against TNFR 1 or 2 on LPS-induced upregulation of Na^+^ current density in different types of neurons**. **(A)** Series of original current recordings from bipolar neurons elicited by step depolarizations in 5 mV increments starting from a holding potential of −77 mV. Analysis of the whole data set for **(B)** bipolar and **(C)** pyramid-shaped cells. (Ba,Ca) Average current–voltage relationships for Na^+^ currents normalized to capacitance recorded from neurons cultured in the presence of microglia and 4 days of exposure to 1 μg LPS and 4 days of either antibody against TNFR 1 or 2. (Bb,Cb) Influence of TNFR 1 and 2 blockage on steady-state inactivation of Na^+^ currents. Solid lines represent fits to the modified Boltzman equation as detailed in Section “Materials and Methods.” (Bc,Cc) Peak Na^+^ current densities of neurons cultured in the presence of microglia and 4 days of exposure to 1 μg LPS and 4 days of either antibody against TNFR 1 or 2 determined at test potentials of 12 mV starting from holding potentials of −77 mV. Note that the Na^+^ current density of bipolar neurons is decreased by both antibodies against TNFR 1 and 2, whereas the Na^+^ current density of pyramid-shaped neurons is only decreased by antibodies against TNFR 2 to control levels. All recordings performed at day 7 in culture after incubations with test substances for 4 days following a preculture of 3 days. Numbers in bar charts indicate numbers of cells recorded from. Database for every column derived from 7 to 15 different preparations. Error bars represent means ± SE, *p < 0.05, **p < 0.01, and ***p < 0.005.

To investigate whether TNF-α is involved in the upregulation of Na^+^ current density by activated microglia and which receptors mediate the effect, we now performed experiments using antibodies against TNF-receptors (TNFR 1 and 2). Cultures obtained from 2- to 4-day postnatal rats were cocultivated with ~15,000 microglia (5%). After 3 days of preincubation, the cultures were exposed to NB medium supplemented with either 1 μg/ml LPS, 1 μg/ml LPS plus 10 μg/ml antibody against TNFR 1, 1 μg/ml LPS plus 10 μg/ml antibody against TNFR 2, or without supplements (control). As shown in Figure 3, we observed significant differences in the Na^+^ current regulation in bipolar and pyramid-shaped neurons. In bipolar neurons, a blockage of both TNFR 1 and 2 led to a decrease of the Na^+^ current density from 69.9 ± 4.5 pA/pF back to control level (in the presence of 5% microglia, all values between 50 and 55 pA/pF). In pyramid-shaped neurons, only a blockage of TNFR 2 led to a decrease of the Na^+^ current density back to control level (5% microglia) of ~60 pA/pF, whereas in the presence of antibodies against TNFR 1, the same elevated Na^+^ current density as in LPS-activated microglia of ~75 pA/pF was observed.

#### Regulatory Effect of IL-18 on the Na^+^ Current Density

**Figure 4:**
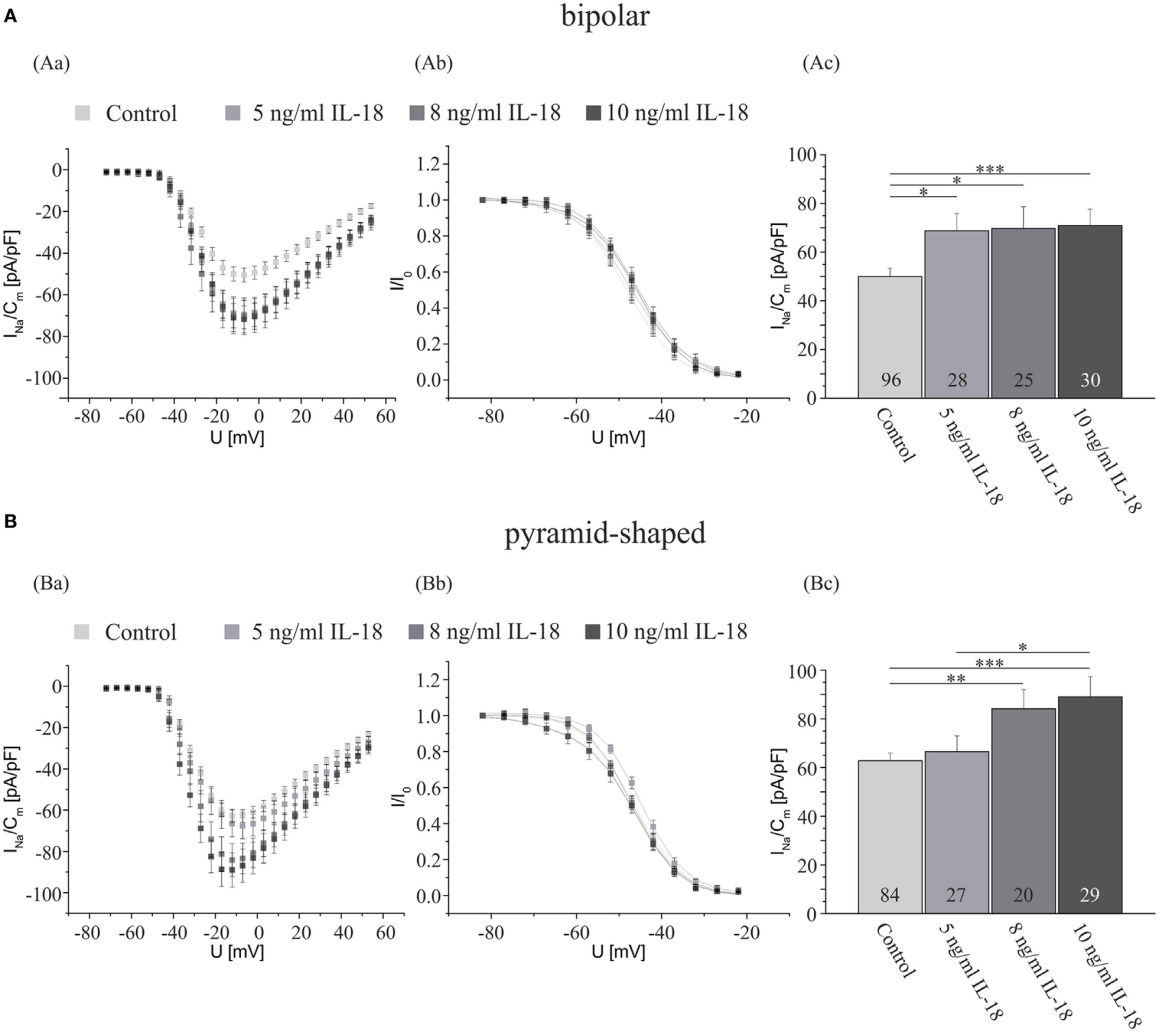
**IL-18 exposure for 4 days increases the Na^+^ current density of cultured hippocampal neurons**. Analysis of the whole data set for **(A)** bipolar and **(B)** pyramid-shaped cells. (Aa,Ba) Average current–voltage relationships for Na^+^ currents normalized to capacitance recorded from neurons cultured in the presence or absence of 5, 8, or 10 ng/ml IL-18 for 4 days. (Ab,Bb) Influence of IL-18 on steady-state inactivation of Na^+^ currents. Solid lines represent fits to the modified Boltzman equation as detailed in Section “Materials and Methods.” (Ac,Bc) Peak Na^+^ current densities of neurons cultured in the presence or absence of 5, 8, or 10 ng/ml IL-18 for 4 days determined at test potentials of −12 mV starting from holding potentials of −77 mV. Note that the Na^+^ current density increases in a dose-dependent manner. The dose–response curve for pyramid-shaped neurons is shifted toward higher concentrations. All recordings performed at day 7 in culture after incubations with test substances for 4 days following a preculture period of 3 days. Numbers in bar charts indicate numbers of cells recorded from. Database for every column derived from 7 to 24 different preparations. Since not all experiments were performed in sister cultures incubated under all four conditions the data for the control cultures in this series of experiments have been pooled. Error bars represent means ± SE, *p < 0.05, **p < 0.01, and ***p < 0.005.

The increases in Na^+^ current density induced by an incubation with TNF-α were smaller than the increase observed after cocultivation with LPS-activated microglia (compare Figure 1 with Figure 2). Furthermore, the blockage of the TNF-α receptors by antibodies did not reduce the Na^+^ currents to the levels obtained in the presence of TGF-β if the microglia were added in cell culture inserts (compare Figures 1Bc,Cc with Figures 3Bc,Cc). We thus considered that other factors released from microglia might additionally contribute to the regulation of I_NavD_. Potential candidates are proinflammatory cytokines of the IL1/18-family. Preliminary data yielded no significant change in the Na^+^ current density in hippocampal neurons incubated with 0.01 ng/ml IL-1α and 0.02 ng/ml IL-6 (data not shown). Since we were not aware of any investigations of IL-18 effects on voltage-activated ion currents we now investigated, whether a preincubation with this interleukin for 4 days affects I_NavD_ in bipolar and hippocampal neurons. Cultures obtained from 2- to 4-day-old postnatal rats were exposed to NB medium supplemented with or without (control) 5, 8, or 10 ng/ml IL-18 after 3 days of preincubation. As shown in Figures 4Ac,Bc, a significant increase in the Na^+^ current density was observed in both, bipolar and pyramid-shaped neurons. While bipolar neurons reached a saturation level at 5 ng/ml IL-18 with an increase of I_NavD_ by 40%, maximal values were observed for pyramid-shaped neurons only at higher concentrations of 8–10 ng/ml with average increases of I_NaVD_ by 40%. These observations suggest cell-type specific different dose–response curves for the regulation of the Na^+^ current density by IL-18.

## Discussion

As Na^+^ currents are essential for the initiation and propagation of neuronal firing, changes in the current density can lead to abnormal neuronal activity. Na^+^ current density can be modulated by small molecular weight neurotransmitters, such as acetylcholine and dopamine by the activation of phosphorylation cascades in a form of short-term plasticity (41). Na^+^ channels may be regulated acutely via the activation of G-proteins (41–43) and over larger time spans by an increase in channel synthesis [see e.g. Ref. (44)].

Apart from release of neurotransmitters out of neurons, astrocytes and microglia are sources of additional neuroactive factors acting at a longer time scale in the CNS [see e.g. Ref. (45)]. For instance, by release of FGF-2 from thyroid hormone-stimulated satellite cells the Na^+^ current density of neurons and thus their excitability can be increased over the course of several days in postnatal, maturing neurons (33). In addition, our research group had previously observed, that LPS-stimulated microglia can increase Na^+^ current density, and that this effect can be mimicked by 100 ng/ml TNF-α (33). Here, we extend these observations showing that the presence of activated microglia in hippocampal cultures effects Na^+^ current densities to some extent differently in bipolar and pyramid-shaped neurons, potentially changing the balance between excitation and inhibition. Thus conditions of neuronal hyper- as well as hypoexcitability under inflammatory conditions could be explained by the release of neuroactive factors from microglia.

### Role of Microglia

Microglia are part of the innate immune system in the CNS and play an important role in inducing and propagating inflammatory signals in response to activation of the peripheral immune system (46) or during brain lesion. TGF-β is a known deactivator of microglia. Kim et al. (47) suggest TGF-β as Phosphatidylinositol 3-Kinase (PI3K) inhibitor, which in turn is linked to the TLR4-mediated cytokine release (48). TGF-β is a 24-kDa protein produced by various types of cells, not only from T and B lymphocytes (49, 50), activated macrophages (51, 52) but also from resident glial cells of the CNS such as astrocytes and microglia (53–55). The effects of TGF-β on cells of the monocyte lineage are multipotential with diverse effects, such as inducing chemotaxis on monocytes and IL-1 production (56). Interestingly, IL-1 and IL-1α induce TGF-β expression in glial cells, which suggests a positive autocrine feedback loop (57). In contrast, TGF-β blocks Interferon-γ (IFN-γ)-induced macrophage activation (58, 59), including the induction of class II major histocompatibility complexes (MHC) antigen (60) and the reduction of macrophagic inhibition of intracellular replication of certain parasites (61). Furthermore, it suppresses proliferation and LPS-induced activation of microglia (62, 63), regulates cell survival (47), and decreases cytokine release, including IL-1, IL-1α, IL-6, TNF-α, and nitric oxide (NO) (37, 62).

Transforming growth factor-β regulates ion channels in microglia. It upregulates delayed rectifier (DR) Kv1.3 K^+^ channel though it shows no effect on inward rectifier (IR) K^+^ channels (64, 65), which could lead to an inhibition of vesicle release by hyperpolarization.

In accordance with previous observations of an inhibitory role of TGF-β on microglia, we here observed (see Figure 1) that the incubation of microglia-enriched cultures with 10 ng/ml TGF-β leads to a decrease of the Na^+^ current density in both, bipolar and pyramid-shaped neurons to values smaller than those observed in control cultures. Although further experiments are needed to further corroborate this hypothesis, our observation suggests that in untreated control cultures, the residual 1–3% microglia release neuroactive factors which influence the Na^+^ current of neurons in their vicinity. LPS stimulates the release of various neuroactive factors from microglia (12). As shown in Figures 1 and 3, we extend our previous findings (33) that the presence of LPS-treated microglia leads to an upregulation of I_NavD_, and show here that a larger increase in the Na^+^ current density can be observed in bipolar compared with pyramid-shaped neurons. Guo et al. (66) linked conditions of chronic pain to the innate immune system of the CNS by the activation of TLRs on microglia, which are activated among others, by LPS. Our findings of the increase of the Na^+^ current density of neurons by LPS-activated microglia support the idea of microglia as a link to hyperalgesia and hyperexcitability.

### Effects of Neuroactive Factors Released from Microglia

Tumor necrosis factor-α is the most prominent cytokine released from microglia. In the brain, it has mostly been associated with leukocyte recruitment through the regulation of adhesion processes and is involved in the activation of glial cells in the process of gliotic scar formation (67). TNF-α belongs to the superfamily of type II transmembrane proteins with intracellular N-terminus (68). It originates in a 26-kDa homotrimeric pro-molecule, which is converted by the metalloprotease TNF-α converting enzyme (TACE) to a diffusible peptide, which forms a non-covalently bound trimer (69) and is secreted from macrophages and monocytes [see e.g. Ref. 70, 71]. The source of TNF-α in the CNS are activated microglia (72). To our knowledge, effects of TNF-α on Na^+^ current densities in central neurons have only been investigated in one study (33). Here, we extend these previous results showing that already an incubation of cultures for 4 days with a concentration of 10 ng/ml elicits maximal effects and that the upregulation of the I_NavD_ is slightly larger in bipolar compared with pyramid-shaped cells. In our previous publication (33), we had observed a significant upregulation after treatment with 100 ng/ml TNF-α in the order of magnitude of 20% using a larger database of about 70 cells recorded under each condition. In the present series of experiments, we recorded from a smaller number of cells under each concentration such that the treatment with 100 ng/ml TNF-α did not yet reach significance. The decline seen with increasing concentrations in the present series of experiments might result from the deleterious effects of higher dosages of TNF-α, which made it increasingly difficult to obtain stable patch clamp recordings from neurons.

Tumor necrosis factor-α binds exclusively to two receptors, TNFR 1 and 2. TNFR 1 is regarded as the primary signaling receptor for systemic TNF-α inflammatory responses (73, 74). TNFR 2 may mediate TNF-α effects in a paracrine or autocrine manner because it is strongly activated by membrane-bound TNF-α (75). Distinct effects of TNFR activation on the TTX-resistant Nav1.8 currents of DRG neurons (76) could be observed.

Our present findings suggest that bipolar neurons are regulated by both TNFR subtypes, while pyramid-shaped neurons are mainly regulated by TNFR 2, suggesting that I_NavD_ in both types of neurons can be regulated differentially by TNF-α.

The observation that the blockage of TNF-α receptors reduced I_NavD_ to a smaller extent than by the inactivation of microglia by TGF-β suggests, that additional cytokines, released by microglia could contribute to I_NavD_-regulation by microglial activation.

Apart from TNF-α, microglia have been shown to release cytokines from the IL-1 family (77). IL-18 is a member of the IL-1 family of proinflammatory cytokines. It is synthesized as an inactive 24-kDa precursor protein that is subsequently cleaved by caspase-1 into its active form (78). The IL-18 receptor (IL-18R) is expressed on a variety of cells, including hypothalamic neurons and murine glia (79, 80).

The effect of cytokines of the IL-1/IL-18 family on Na^+^ currents of hippocampal neurons has to our knowledge not been investigated. Interestingly, both interleukins and TNF-α are implicated in sleep regulation (81). Both enhance non-rapid eye movement sleep (NREMS). While the exact mechanism is currently unknown, it might be possible that an alteration of neuronal excitability might be involved in the enhancement of the NREMS phase. Interestingly though, a variety of cytokines has been linked to the increase of NREM sleep during inflammation, including IL-1, IL-18, and TNF-α (81–83).

Our present results suggest that IL-18 may additionally regulate I_NavD_ in central neurons. Differences in the dose–response curves observed in bipolar and pyramid-shaped neurons suggest that bipolar cells might already respond to lower activations of microglial cells releasing less IL-18. Our findings that bipolar inhibitory neurons are regulated more strongly by IL-18 and TNF-α and subsequently lead to an overall hypoexcitability of the neural network and may explain symptoms such as dizziness or an increase in NREM sleep during infectious diseases.

Tumor necrosis factor-α not only exerts long-term effects but is also capable of an acute p38-mediated modulation of Tetrodotoxin-resistant (TTX-R) Na^+^ channels in mouse sensory neurons (84). During phosphorylation, changes in the voltage-dependence of inactivation have been observed by Chizhmakov et al. and Franceschetti et al. (85, 86). For both cytokines tested here, we observed no significant changes in the voltage-dependence of the inactivation of the Na^+^ currents. This indicates that the increased Na^+^ current densities might rather be caused by increased ion channel densities instead via short-term regulatory mechanisms such as phosphorylation (87).

Taken together, this study presents evidence that two major cytokines, namely TNF-α and IL-18, released from microglia activated by pathological events such as CNS injury or inflammation (6, 14, 15, 28, 36, 88), not only effect, e.g., cell survival (89) and death (90), DNA-synthesis (91), cell proliferation (92, 93), cell differentiation (89, 94), and neuronal cell fate in embryonic neural progenitor cells (NPC) (95) but also in addition upregulate Na^+^ current density and thus excitability in hippocampal neurons. The differential regulation of the Na^+^ current densities in bipolar and pyramid-shaped neurons can lead to an imbalance between inhibition and excitation in the neural network. At the network level, this could lead to an overall neuronal hypoexcitability, which might be an explanation for the symptom of dizziness during neuroinflammation (96). These findings are in line with the results of Richter et al. that TNF-α reduces the amplitude of cortical spreading depression (CSD) by the activation of TNFR 2 in cortical inhibitory neurons (35). The differentiation by the TNFR subtypes enables an asymmetric increase of Na^+^ currents by the same concentration of TNF-α and might be an additional explanation that different concentrations of TNF-α can lead to bidirectional effects on cortical network activity (97). Another mechanism of differential Na^+^ current density regulation is the dose-dependent effect of IL-18. At low concentrations, it mainly affects the Na^+^ current density of inhibitory interneurons, while at higher concentrations (8–10 ng) also pyramid-shaped neurons are affected. This finding could contribute to the origin of a general inhibition of cortical networks followed by a general excitation with a further increase of the neuroinflammation. Such a mechanism, leading with a delay of several days after the onset of the infection first to dizziness and then to epileptic seizures might also explain the neurological symptoms, which occurred during immune reactions in 20% of the patients over the course of the recent outbreak of a Shiga-toxin producing *E. coli* infection (98).

## Ethics Statement

Animals were bred in the animal house of the Faculty for Medicine at the Ruhr-University and all procedures adhered to the German animal protection law.

## Author Contributions

ID designed the experiments and participated in data analysis, interpretation, and writing of the manuscript. LK acquired and analyzed most of the data, participated in the conception of the experiments, prepared the figures, and drafted the article. BI contributed to the conception of the experiments, some of the data and tutoring of LK during his Master’s thesis, which contains parts identical to the present publication. All authors approved the version to be published.

## Conflict of Interest Statement

The authors declare that the research was conducted in the absence of any commercial or financial relationships that could be construed as a potential conflict of interest.

## References

[1] Canolle B Masmejean F Melon C Nieoullon A Pisano P Lortet S. Glial soluble factors regulate the activity and expression of the neuronal glutamate transporter EAAC1: implication of cholesterol. J Neurochem (2004) 88:1521–32. 10.1016/j.jphysparis.2005.12.048 15009653

[2] Potthoff O Dietzel ID. Thyroid hormone regulates Na+ currents in cultured hippocampal neurons from postnatal rats. Proc Biol Sci (1997) 264:367–73. 10.1098/rspb.1997.0053 9107052 PMC1688264

[3] Hoffmann G Dietzel ID. Thyroid hormone regulates excitability in central neurons from postnatal rats. Neuroscience (2004) 125:369–79. 10.1016/j.neuroscience.2004.01.047 15062980

[4] Niederkinkhaus V Marx R Hoffmann G Dietzel ID Thyroid hormone (T(3))-induced up-regulation of voltage-activated sodium current in cultured postnatal hippocampal neurons requires secretion of soluble factors from glial cells. Mol Endocrinol (2009) 23:1494–504. 10.1210/me.2009-0132 19460859 PMC2737559

[5] Graeber MB. Changing face of microglia. Science (2010) 330:783–8. 10.1126/science.1190929 21051630

[6] Giulian D. Ameboid microglia as effectors of inflammation in the central nervous system. J Neurosci Res (1987) 18(155–171):132–3. 10.1002/jnr.490180123 3500323

[7] Streit WJ Graeber MB Kreutzberg GW. Functional plasticity of microglia: a review. Glia (1988) 1:301–7. 10.1002/glia.440010502 2976393

[8] Nimmerjahn A Kirchhoff F Helmchen F. Resting microglial cells are highly dynamic surveillants of brain parenchyma in vivo. Neuroforum (2005) 11:95–6. 10.1126/science.1110647 15831717

[9] Stence N Waite M Dailey ME. Dynamics of microglial activation: a confocal time-lapse analysis in hippocampal slices. Glia (2001) 33:256–66. 10.1002/1098-1136(200103)33:3<256:AID-GLIA1024>3.0.CO;2-J 11241743

[10] Block ML Zecca L Hong J-S. Microglia-mediated neurotoxicity: uncovering the molecular mechanisms. Nat Rev Neurosci (2007) 8:57–69. 10.1038/nrn2038 17180163

[11] Aloisi F Ria F Adorini L. Regulation of T-cell responses by CNS antigen-presenting cells: different roles for microglia and astrocytes. Immunol Today (2000) 21:141–8. 10.1016/S0167-5699(99)01512-1 10689302

[12] Nakamura Y Si QS Kataoka K. Lipopolysaccharide-induced microglial activation in culture: temporal profiles of morphological change and release of cytokines and nitric oxide. Neurosci Res (1999) 35:95–100. 10.1016/S0168-0102(99)00071-1 10616913

[13] Jack CS Arbour N Manusow J Montgrain V Blain M McCrea E TLR signaling tailors innate immune responses in human microglia and astrocytes. J Immunol (2005) 175:4320–30. 10.4049/jimmunol.175.7.4320 16177072

[14] Olson JK Miller SD. Microglia initiate central nervous system innate and adaptive immune responses through multiple TLRs. J Immunol (2004) 173:3916–24. 10.4049/jimmunol.173.6.3916 15356140

[15] Lehnardt S. Innate immunity and neuroinflammation in the CNS: the role of microglia in toll-like receptor-mediated neuronal injury. Glia (2010) 58:253–63. 10.1002/glia.20928 19705460

[16] Nakajima K Kohsaka S. Microglia: activation and their significance in the central nervous system. J Biochem (2001) 130:169–75. 10.1093/oxfordjournals.jbchem.a002969 11481032

[17] Gould HJ Gould TN England JD Paul D Liu ZP Levinson SR. A possible role for nerve growth factor in the augmentation of sodium channels in models of chronic pain. Brain Res (2000) 854:19–29. 10.1016/S0006-8993(99)02216-7 10784102

[18] Chen X Pang RP Shen KF Zimmermann M Xin WJ Li YY TNF-alpha enhances the currents of voltage gated sodium channels in uninjured dorsal root ganglion neurons following motor nerve injury. Exp Neurol (2011) 227:279–86. 10.1016/j.expneurol.2010.11.017 21145890

[19] Djouhri L Lawson SN. Changes in somatic action potential shape in guinea-pig nociceptive primary afferent neurones during inflammation in vivo. J Physiol (1999) 520:565–76. 10.1111/j.1469-7793.1999.t01-1-00565.x 10523423 PMC2269587

[20] Fanger GR Erhardt P Cooper GM Maue RA ras-independent induction of rat brain type II sodium channel expression in nerve growth factor-treated PC12 cells. J Neurochem (1993) 61:1977–80. 10.1111/j.1471-4159.1993.tb09844.x 8229007

[21] He XH Zang Y Chen X Pang RP Xu JT Zhou X TNF-alpha contributes to up-regulation of Nav1.3 and Nav1.8 in DRG neurons following motor fiber injury. Pain (2010) 151:266–79. 10.1016/j.pain.2010.06.005 20638792

[22] Tamura R Nemoto T Maruta T Onizuka S Yanagita T Wada A Up-regulation of NaV1.7 sodium channels expression by tumor necrosis factor-α in cultured bovine adrenal chromaffin cells and rat dorsal root ganglion neurons. Anesth Analg (2014) 118:318–24. 10.1213/ANE.0000000000000085 24445633

[23] Dib-Hajj SD Cummins TR Black JA Waxman SG. Sodium channels in normal and pathological pain. Annu Rev Neurosci (2010) 33:325–47. 10.1146/annurev-neuro-060909-153234 20367448

[24] McGowan E Hoyt SB Li X Lyons KA Abbadie C A peripherally acting Nav1.7 sodium channel blocker reverses hyperalgesia and allodynia on rat models of inflammatory and neuropathic pain. Anesth Analg (2009) 109:951–8. 10.1213/ane.0b013e3181b01b02 19690272

[25] Joshi SK Mikusa JP Hernandez G Baker S Shieh CC Neelands T Involvement of the TTX-resistant sodium channel Nav 1.8 in inflammatory and neuropathic, but not post-operative, pain states. Pain (2006) 123:75–82. 10.1016/j.pain.2006.02.011 16545521

[26] Kerr BJ Souslova V McMahon SB Wood JN. A role for the TTX-resistant sodium channel Nav 1.8 in NGF-induced hyperalgesia, but not neuropathic pain. Neuroreport (2001) 12:3077–80. 10.1097/00001756-200110080-00019 11568640

[27] Lai J Porreca F Hunter JC Gold MS. Voltage-gated sodium channels and hyperalgesia. Annu Rev Pharmacol Toxicol (2004) 44:371–97. 10.1146/annurev.pharmtox.44.101802.121627 14744251

[28] Vezzani A Granata T. Brain inflammation in epilepsy: experimental and clinical evidence. Epilepsia (2005) 46:1724–43. 10.1111/j.1528-1167.2005.00298.x 16302852

[29] Singhi P. Infectious causes of seizures and epilepsy in the developing world. Dev Med Child Neurol (2011) 53:600–9. 10.1111/j.1469-8749.2011.03928.x 21518343

[30] Lancman ME Morris HH. Epilepsy after central nervous system infection: clinical characteristics and outcome after epilepsy surgery. Epilepsy Res (1996) 25:285–90. 10.1016/S0920-1211(96)00086-1 8956928

[31] Devinsky O Vezzani A Najjar S De Lanerolle NC Rogawski MA Glia and epilepsy: excitability and inflammation. Trends Neurosci (2013) 36:174–84. 10.1016/j.tins.2012.11.008 23298414

[32] Najjar S Pearlman D Miller DC Devinsky O. Refractory epilepsy associated with microglial activation. Neurologist (2011) 17:249–54. 10.1097/NRL.0b013e31822aad04 21881466

[33] Igelhorst BA Niederkinkhaus V Karus C Lange MD Dietzel ID. Regulation of neuronal excitability by release of proteins from glial cells. Philos Trans R Soc Lond B Biol Sci (2015) 370:20140194. 10.1098/rstb.2014.0194 26009773 PMC4455763

[34] Hughes JR Drachman DA. Dizziness, epilepsy and the EEG. Dis Nerv Syst (1977) 38:431–5. 862501

[35] Richter F Lütz W Eitner A Leuchtweis J Lehmenkühler A Schaible HG. Tumor necrosis factor reduces the amplitude of rat cortical spreading depression in vivo. Ann Neurol (2014) 76:43–53. 10.1002/ana.24176 24798682

[36] Hanisch UK. Microglia as a source and target of cytokines. Glia (2002) 40:140–55. 10.1002/glia.10161 12379902

[37] Lodge PA Sriram S Regulation of microglial activation by TGF-β, IL-10 and CSF-1. J Leukoc Biol (1996) 60:502–508. 8864135 10.1002/jlb.60.4.502

[38] Brewer GJ Cotman CW. Survival and growth of hippocampal neurons in defined medium at low density: advantages of a sandwich culture technique or low oxygen. Brain Res (1989) 494:65–74. 10.1016/0006-8993(89)90144-3 2765923

[39] McCarthy K De Vellis J Preparation of seperate astroglial and oligodendroglial cultures from rat cerebral tissue. J Cell Biol (1980) 85:890–902. 10.1083/jcb.85.3.890 6248568 PMC2111442

[40] Kleinsimlinghaus K Marx R Serdar M Bendix I Dietzel ID. Strategies for repair of white matter: influence of osmolarity and microglia on proliferation and apoptosis of oligodendrocyte precursor cells in different basal culture media. Front Cell Neurosci (2013) 7:277. 10.3389/fncel.2013.00277 24421756 PMC3872727

[41] Cantrell AR Catterall WA. Neuromodulation of Na^+^ channels: an unexpected form of cellular plasticity. Nat Rev Neurosci (2001) 2:397–407. 10.1038/35077553 11389473

[42] Brown AM Birnbaumer L Ionic channels and their regulation by G protein subunits. Annu Rev Physiol (1990) 52:197–213. 10.1146/annurev.ph.52.030190.001213 1691904

[43] Wickman KD Clapham DE G-protein regulation of ion channels. Curr Opin Neurobiol (1995) 5:278–85. 10.1016/0959-4388(95)80039-5 7580149

[44] Brodie C Sampson SR. Characterization of thyroid hormone effects on Na channel synthesis in cultured skeletal myotubes: role of Ca^2+^. Endocrinology (1989) 125:842–9. 10.1210/endo-125-2-842 2546750

[45] Martin DL. Synthesis and release of neuroactive substances by glial cells. Glia (1992) 5:81–94. 10.1002/glia.440050202 1349588

[46] Nguyen MD Julien J-P Rivest S. Innate immunity: the missing link in neuroprotection and neurodegeneration? Nat Rev Neurosci (2002) 3:216–27. 10.1038/nrn752 11994753

[47] Kim W-K Hwang S-Y Oh E-S Piao HZ Kim K-W Han I-O TGF-beta1 represses activation and resultant death of microglia via inhibition of phosphatidylinositol 3-kinase activity. J Immunol (2004) 172:7015–23. 10.4049/jimmunol.172.11.7015 15153523

[48] Ojaniemi M Glumoff V Harju K Liljeroos M Vuori K Hallman M. Phosphatidylinositol 3-kinase is involved in toll-like receptor 4-mediated cytokine expression in mouse macrophages. Eur J Immunol (2003) 33:597–605. 10.1002/eji.200323376 12616480

[49] Kehrl JH Roberts AB Wakefield LM Jakowlew S Sporn MB Fauci AS. Transforming growth factor beta is an important immunomodulatory protein for human B lymphocytes. J Immunol (1986) 137:3855–60. 2878044

[50] Kehrl JH Wakefield LM Roberts AB Jakowlew S Alvarez-Mon M Derynck R Production of transforming growth factor beta by human T lymphocytes and its potential role in the regulation of T cell growth. J Exp Med (1986) 163:1037–50. 10.1084/jem.163.5.1037 2871125 PMC2188095

[51] Assoian RK Fleurdelys BE Stevenson HC Miller PJ Madtes DK Raines EW Expression and secretion of type beta transforming growth factor by activated human macrophages. Proc Natl Acad Sci U S A (1987) 84:6020–4. 10.1073/pnas.84.17.6020 2888109 PMC298999

[52] Grotendorst GR Smale G Pencev D. Production of transforming growth factor beta by human peripheral blood monocytes and neutrophils. J Cell Physiol (1989) 140:396–402. 10.1002/jcp.1041400226 2745570

[53] Wahl SM Allen JB McCartney-Francis N Morganti-Kossmann MC Kossmann T Ellingsworth L Macrophage- and astrocyte-derived transforming growth factor beta as a mediator of central nervous system dysfunction in acquired immune deficiency syndrome. J Exp Med (1991) 173:981–91. 10.1084/jem.173.4.981 2007861 PMC2190818

[54] Saad B Constam DB Ortmann R Moos M Fontana A Schachner M. Astrocyte-derived TGF-beta 2 and NGF differentially regulate neural recognition molecule expression by cultured astrocytes. J Cell Biol (1991) 115:473–84. 10.1083/jcb.115.2.473 1717486 PMC2289154

[55] Constam DB Philipp J Malipiero UV ten Dijke P Schachner M Fontana A. Differential expression of transforming growth factor-beta 1, -beta 2, and -beta 3 by glioblastoma cells, astrocytes, and microglia. J Immunol (1992) 148:1404–10. 1538124

[56] Wahl SM Hunt DA Wakefield LM McCartney-Francis N Wahl LM Roberts AB Transforming growth factor type beta induces monocyte chemotaxis and growth factor production. Proc Natl Acad Sci U S A (1987) 84:5788–92. 10.1073/pnas.84.16.5788 2886992 PMC298948

[57] da Cunha A Jefferson JA Jackson RW Vitković L. Glial cell-specific mechanisms of TGF-beta 1 induction by IL-1 in cerebral cortex. J Neuroimmunol (1993) 42:71–85. 10.1016/0165-5728(93)90214-J 8423208

[58] Tsunawaki S Sporn M Ding A Nathan C. Deactivation of macrophages by transforming growth factor-beta. Nature (1988) 334:260–2. 10.1038/334260a0 3041283

[59] Ding A Nathan CF Graycar J Derynck R Stuehr DJ Srimal S. Macrophage deactivating factor and transforming growth factors-beta 1 -beta 2 and -beta 3 inhibit induction of macrophage nitrogen oxide synthesis by IFN-gamma. J Immunol (1990) 145:940–4. 2115549

[60] Czarniecki CW Chiu HH Wong GH McCabe SM Palladino MA. Transforming growth factor-beta 1 modulates the expression of class II histocompatibility antigens on human cells. J Immunol (1988) 140:4217–23. 3131428

[61] Barral-Netto M Barral A Brownell CE Skeiky YA Ellingsworth LR Twardzik DR Transforming growth factor-beta in leishmanial infection: a parasite escape mechanism. Science (1992) 257:545–8. 10.1126/science.1636092 1636092

[62] Suzumura A Yamamoto H Marunouchi T Transforming growth factor-beta suppresses activation and proliferation of microglia in vitro. J Immunol (1993) 151:2150–8. 8345199

[63] Jones LL Kreutzberg GW Raivich G Transforming growth factor β’s 1, 2 and 3 inhibit proliferation of ramified microglia on an astrocyte monolayer. Brain Res (1998) 795:301–6. 10.1016/S0006-8993(98)00325-4 9622658

[64] Schilling T Nitsch R Heinemann U Haas D Eder C. Astrocyte-released cytokines induce ramification and outward K^+^ channel expression in microglia via distinct signalling pathways. Eur J Neurosci (2001) 14:463–73. 10.1046/j.0953-816X.2001.01661.x 11553296

[65] Schilling T Quandt FN Cherny VV Zhou W Heinemann U Decoursey TE Upregulation of Kv1.3 K(+) channels in microglia deactivated by TGF-beta. Am J Physiol Cell Physiol (2000) 279:C1123–34. 11003593 10.1152/ajpcell.2000.279.4.C1123

[66] Guo L-H Schluesener HJ. The innate immunity of the central nervous system in chronic pain: the role of Toll-like receptors. Cell Mol Life Sci (2007) 64:1128–36. 10.1007/s00018-007-6494-3 17440679 PMC11136004

[67] Feuerstein GZ Liu T Barone FC. Cytokines, inflammation, and brain injury: role of tumor necrosis factor-alpha. Cerebrovasc Brain Metab Rev (1994) 6:341–60. 7880718

[68] Forge A Wright T The molecular architecture of the inner ear. Brit Med Bull (2002) 63:5–24. 10.1016/S0968-0004(01)01995-8 12324381

[69] Tang P Hung MC Klostergaard J. Human pro-tumor necrosis factor is a homotrimer. Biochemistry (1996) 35:8216–25. 10.1021/bi952182t 8679576

[70] Takemura R Werb Z. Secretory products of macrophages and their physiological functions. Am J Physiol (1984) 246:C1–9. 6364825 10.1152/ajpcell.1984.246.1.C1

[71] Kornbluth RS Edgington TS. Tumor necrosis factor production by human monocytes is a regulated event: induction of TNF-alpha-mediated cellular cytotoxicity by endotoxin. J Immunol (1986) 137:2585–91. 3760568

[72] Hetier E Ayala J Bousseau A Prochiantz A Amoeboid microglial cells and not astrocytes synthesize TNF-a in Swiss mouse brain cell cultures. Eur J Neurosci (1990) 2:762–8. 10.1111/j.1460-9568.1990.tb00466.x 12106276

[73] Rothe J Lesslauer W Lötscher H Lang Y Koebel P Köntgen F Mice lacking the tumour necrosis factor receptor 1 are resistant to TNF-mediated toxicity but highly susceptible to infection by *Listeria monocytogenes*. Nature (1993) 364:798–802. 10.1038/364798a0 8395024

[74] Pfeffer K Matsuyama T Kündig TM Wakeham A Kishihara K Shahinian A Mice deficient for the 55 kd tumor necrosis factor receptor are resistant to endotoxic shock, yet succumb to *L. monocytogenes* infection. Cell (1993) 73:457–67. 10.1016/0092-8674(93)90134-C 8387893

[75] Haas E Grell M Wajant H Scheurich P. Continuous autotropic signaling by membrane-expressed tumor necrosis factor. J Biol Chem (1999) 274:18107–12. 10.1074/jbc.274.25.18107 10364265

[76] Leo M Argalski S Schäfers M Hagenacker T. Modulation of voltage-gated sodium channels by activation of tumor necrosis factor receptor-1 and receptor-2 in small DRG neurons of rats. Mediators Inflamm (2015) 2015:124942. 10.1155/2015/124942 26504355 PMC4609494

[77] Giulian D Baker TJ Shih LC Lachman LB. Interleukin 1 of the central nervous system is produced by ameboid microglia. J Exp Med (1986) 164:594–604. 10.1084/jem.164.2.594 3487617 PMC2188228

[78] Gracie JA Robertson SE McInnes IB. Interleukin-18. J Leukoc Biol (2003) 73:213–24. 10.1189/jlb.0602313 12554798

[79] Wheeler RD Brough D Le Feuvre RA Takeda K Iwakura Y Luheshi GN Interleukin-18 induces expression and release of cytokines from murine glial cells: interactions with interleukin-1β. J Neurochem (2003) 85:1412–20. 10.1046/j.1471-4159.2003.01787.x 12787061

[80] Wheeler RD Culhane AC Hall MD Pickering-Brown S Rothwell NJ Luheshi GN. Detection of the interleukin 18 family in rat brain by RT-PCR. Mol Brain Res (2000) 77:290–3. 10.1016/S0169-328X(00)00069-3 10837926

[81] Krueger JM Obál F Fang J Kubota T Taishi P. The role of cytokines in physiological sleep regulation. Ann N Y Acad Sci (2001) 933:211–21. 10.1111/j.1749-6632.2001.tb05826.x 12000022

[82] Opp MR. Cytokines and sleep. Sleep Med Rev (2005) 9:355–64. 10.1016/j.smrv.2005.01.002 16102986

[83] Imeri L Opp MR. How (and why) the immune system makes us sleep. Nat Rev Neurosci (2009) 10:199–210. 10.1038/nrn2576 19209176 PMC2839418

[84] Jin X Gereau RW. Acute p38-mediated modulation of tetrodotoxin-resistant sodium channels in mouse sensory neurons by tumor necrosis factor-alpha. J Neurosci (2006) 26:246–55. 10.1523/JNEUROSCI.3858-05.2006 16399694 PMC6674296

[85] Chizhmakov IV Klee MR. The action of a phorbol ester on voltage-dependent parameters of the sodium current in isolated hippocampal neurons. Neuroscience (1994) 59:285–90. 10.1016/0306-4522(94)90596-7 8008192

[86] Franceschetti S Taverna S Sancini G Panzica F Lombardi R Avanzini G. Protein kinase C-dependent modulation of Na+ currents increases the excitability of rat neocortical pyramidal neurones. J Physiol (2000) 528(Pt 2):291–304. 10.1111/j.1469-7793.2000.00291.x 11034619 PMC2270127

[87] Hudmon A Choi J-S Tyrrell L Black JA Rush AM Waxman SG Phosphorylation of sodium channel Na(v)1.8 by p38 mitogen-activated protein kinase increases current density in dorsal root ganglion neurons. J Neurosci (2008) 28:3190–201. 10.1523/JNEUROSCI.4403-07.2008 18354022 PMC6670703

[88] Yatsiv I Morganti-Kossmann MC Perez D Dinarello CA Novick D Rubinstein M Elevated intracranial IL-18 in humans and mice after traumatic brain injury and evidence of neuroprotective effects of IL-18-binding protein after experimental closed head injury. J Cereb Blood Flow Metab (2002) 22:971–8. 10.1097/00004647-200208000-00008 12172382

[89] Coma G Peña R Blanco J Rosell A Borras FE Esté JA Treatment of monocytes with interleukin (IL)-12 plus IL-18 stimulates survival, differentiation and the production of CXC chemokine ligands (CXCL)8, CXCL9 and CXCL10. Clin Exp Immunol (2006) 145:535–44. 10.1111/j.1365-2249.2006.03145.x 16907924 PMC1809701

[90] Rath P Aggarwal B. TNF-induced signaling in apoptosis. J Clin Immunol (1999) 19:350–64. 10.1023/A:1020546615229 10634209

[91] Rolfe M James NH Roberts RA Tumour necrosis factor α (TNFα) suppresses apoptosis and induces DNA synthesis in rodent hepatocytes: a mediator of the hepatocarcinogenicity of peroxisome proliferators? Carcinogenesis (1997) 18:2277–80. 10.1093/carcin/18.11.2277 9395232

[92] Amrani Y Panettieri RA Frossard N Bronner C. Activation of the TNF alpha-p55 receptor induces myocyte proliferation and modulates agonist-evoked calcium transients in cultured human tracheal smooth muscle cells. Am J Respir Cell Mol Biol (1996) 15:55–63. 10.1165/ajrcmb.15.1.8679222 8679222

[93] French AR Holroyd EB Yang L Kim S Yokoyama WM. IL-18 acts synergistically with IL-15 in stimulating natural killer cell proliferation. Cytokine (2006) 35:229–34. 10.1016/j.cyto.2006.08.006 17052916

[94] Lam J Takeshita S Barker JE Kanagawa O Ross FP Teitelbaum SL TNF-α induces osteoclastogenesis by direct stimulation of macrophages exposed to permissive levels of RANK ligand. J Clin Invest (2000) 106:1481–8. 10.1172/JCI11176 11120755 PMC387259

[95] Liu Y-P Lin H-I Tzeng S-F Tumor necrosis factor-α and interleukin-18 modulate neuronal cell fate in embryonic neural progenitor culture. Brain Res (2005) 1054:152–8. 10.1016/j.brainres.2005.06.085 16054598

[96] Lozano D Gonzales-Portillo GS Acosta S de la Pena I Tajiri N Kaneko Y Neuroinflammatory responses to traumatic brain injury: etiology, clinical consequences, and therapeutic opportunities. Neuropsychiatr Dis Treat (2015) 11:97–106. 10.2147/NDT.S65815 25657582 PMC4295534

[97] Yuhas Y Weizman A Ashkenazi S. Bidirectional concentration-dependent effects of tumor necrosis factor alpha in Shigella dysenteriae-related seizures. Infect Immun (2003) 71:2288–91. 10.1128/IAI.71.4.2288-2291.2003 12654859 PMC152059

[98] Magnus T Röther J Simova O Meier-Cillien M Repenthin J Möller F The neurological syndrome in adults during the 2011 northern German *E. coli* serotype O104:H4 outbreak. Brain (2012) 135:1850–9. 10.1093/brain/aws090 22539260

